# Quantification of atropine sulphate monohydrate and obidoxime dichloride in two‐chamber autoinjectors for accessing uniformity of dosage

**DOI:** 10.1002/ansa.202200028

**Published:** 2022-11-12

**Authors:** Iva Spreitzer, Paniz Morawej, Richard Wosolsobe, Rainer Stinzl, Judith Wackerlig

**Affiliations:** ^1^ Department of Pharmaceutical Sciences Faculty of Life Sciences University of Vienna Vienna Austria; ^2^ Military Pharmacy Section Medical Division Federal Ministry of Defence Vienna Austria; ^3^ Armaments and Defence Technology Agency Vienna Austria

**Keywords:** atropine sulphate monohydrate, autoinjector, obidoxime dichloride, uniformity of dosage unit

## Abstract

In the treatment of organophosphate poisoning atropine sulphate monohydrate (AT) and obidoxime dichloride (OB) play a vital role. Currently, the Austrian Armed Forces use the DOUBLEPEN OA two‐chamber autoinjector (ChemProtect) to administer these two drugs. The autoinjector is a part of military standard equipment as a “Basic CBRN‐First Aid Kit” and contains OB and AT with a declared concentration of 220 mg/2 ml and 2 mg/2 ml, respectively. Especially in the two‐chamber autoinjectors, it is highly possible that not all the content of the antidote solution is administered when the autoinjector is triggered. The purpose of the study was to analyze one hundred DOUBLEPEN OA autoinjectors from two different production batches (1707068 and 1707067) for volume loss, drug content and uniformity of dosage unit. Uniformity of dosage units, assessed by the content uniformity method (Chapter 2.9.40 of the European Pharmacopeia), requires the calculation of an acceptable value to quantify the uniformity of the drug product. An acceptance value for the first 10 dosage units of 15.0% or below is considered acceptable. The loss of volume was calculated by determining the density and mass of the solution after triggering the autoinjector. A quantitative high‐performance liquid chromatography method has been developed and in‐house validated for the determination of the content of two drugs. According to International Council for Harmonisation guidelines, the analytical method was proven to be accurate and repeatable. The obtained results show that the average loss of volume after injection was 5%, and the average content of OB and AT for batch 1707068, was 216.5 and 1.9 mg, while for batch 1707067 it was 224.2 and 2.0 mg, respectively. Although the loss of volume and content were observed, the calculated acceptance value for both production batches met the requirements of uniformity of dosage unit by the European Pharmacopeia.

## 1 INTRODUCTION

Poisoning by organophosphate (OP) nerve agents is a life‐threatening peril. OP compounds such as sarin, soman, tabun or VX are inhibitors of the enzyme acetylcholinesterase (AChE) preventing the breakdown of acetylcholine (ACh). During OP poisoning, immediate evaluation and treatment are important since the symptoms are developing rapidly leading to death by respiratory paralysis.^1^, ^2^, ^3^ A combination of atropine sulphate monohydrate (AT) and oximes is recommended to counteract the effect of the cholinergic crisis.^4^ AT acts as an antagonist on the muscarine receptor, counteracting the excessive accumulation of ACh. Oximes such as obidoxime dichloride (OB) reactivate the AChE reversing the effect of the nerve agents.^5^ Special autoinjectors containing antidote solutions are provided as the emergency treatment by self‐application or buddy aid in military standard equipment.^6^ By applying an autoinjector the drug absorption is faster due to the large area of administration compared with manual intramuscular injection.^6^ The precise administration of the antidotes is of great importance since AT and OB are themselves highly toxic substances. The initial doses of AT and OB are 2 mg and 220 mg for intramuscular or intravenous injection, respectively.^1^, ^4^ The procedure should be repeated if needed.

**FIGURE 1:**
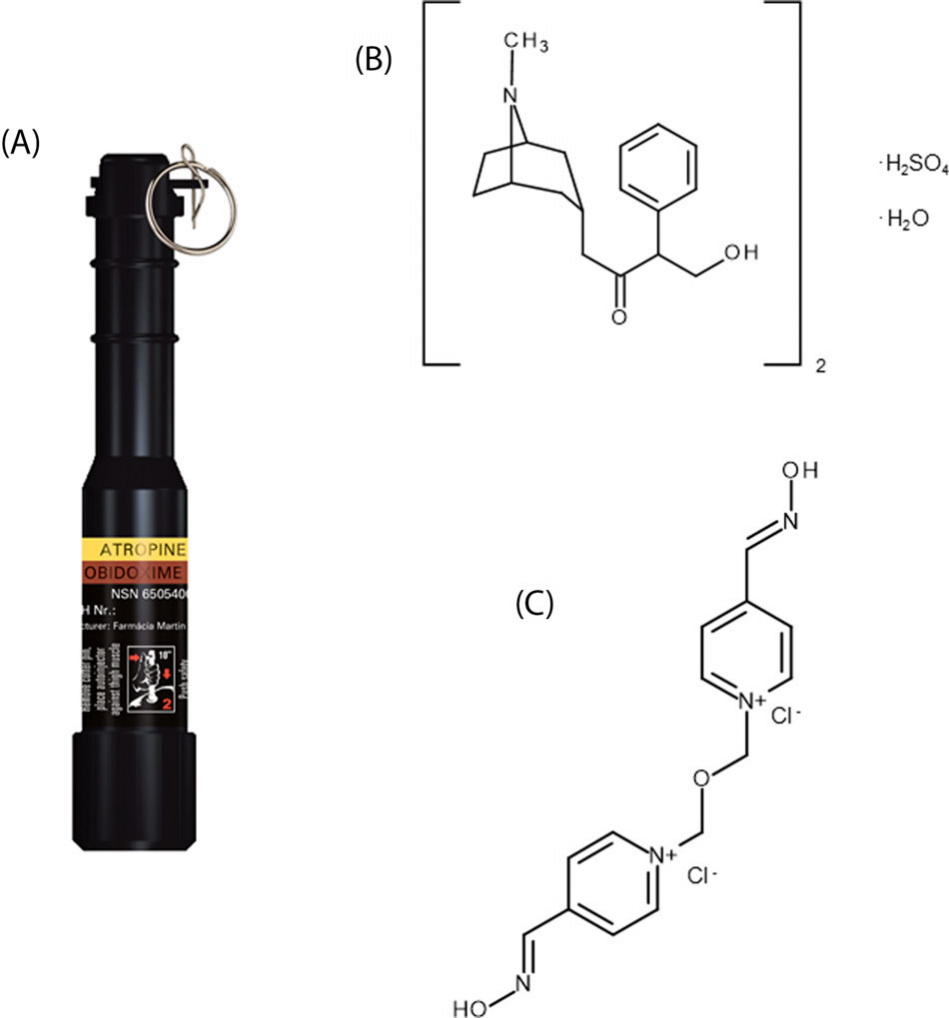
DOUBLEPEN OA two‐chamber autoinjector (ChemProtect) (A)^7^, chemical structures of atropine sulphate monohydrate (B) and obidoxime dichloride (C)

The DOUBLEPEN OA (ChemProtect, Czech Republic) (Figure 1), a two‐chamber autoinjector system, contains individual AT and OB dilutions as antidotes and is in military use by the Austrian Armed Forces as a part of the “Basic CBRN‐First Aid Kit” (CBRN stands for chemical, biological, radiological and nuclear). The autoinjector is a semiautomatic device for first aid in the case of poisoning with AChE inhibitors from the group of OP compounds. It is designed for self‐application or buddy aid intended for single‐dose delivery. The autoinjector is composed of a black plastic body with two rings for easy recognition during the night, a metal safety pin large enough to allow manipulation in gloves, safety fuse, injecting mechanism, and a two‐chamber reservoir. The upper chamber contains AT (2 mg/2 ml), and the lower chamber contains OB (220 mg/2 ml). Injecting mechanism is activated after pulling out the safety pin which releases the spring inside of the autoinjector and shoots the needle through the two‐chambered reservoir. The AT and OB solutions are delivered intramuscularly to the back muscles of the thigh and can penetrate several layers of clothing. Especially in two‐chamber autoinjectors, it is highly possible that not all the content is administered. The reason for this problem could lie in its injection mechanism. After the autoinjector is triggered, the needle is inserted from the upper chamber through the lower one. During this process, the loss of injected volume may occur which may result in the administration of different concentrations of antidote solution as reported.

Most of the work in the field of autoinjectors for the treatment of OP poisoning focuses on the assessment of the efficacy and pharmacokinetics,^8^, ^9^ developing a new antidote formulation^1^ and determining the stability of the antidote solution.^1^, ^10^ The two‐chamber autoinjectors containing a combination of AT in one chamber and the other chamber pralidoxime or bispyridinium oximes (OB and HI 6) were already tested for effective treatment of OP nerve agent poisoning. Results have shown when such drug combination is administered together it did not reduce the overall absorption of antidote solution.^1^, ^3^, ^11^, ^12^ Joosen et al.^9^ have tested the efficacy of a combined AT/OB formulation of Trobigard autoinjector against sarin at two dose levels in a guinea pig model. The results have shown that the pharmacokinetics of AT and OB were proportional to human equivalent doses, and only a small increase in heart rate was observed as a side effect.^9^ However, the efficacy and safety of preparation require a reliable investigation of content and loss after application and not only the pharmacological effect.

In the literature, several analytical methods have been reported for the quantitative determination of AT and OB in antidote solutions.^1^, ^3^, ^13^, ^14^, ^15^, ^16^, ^17^, ^18^ Reported methods require complicated and time‐consuming sample preparation, determination of only one component, peak tailing, need for the internal standard and interference during chromatography.

The purpose of the study is to assess the accuracy of the DOUBLEPEN OA autoinjector. The content and volume loss after its application have not been tested yet and to the best of our knowledge, this represents the first study of that kind. A simple and rapid high‐performance liquid chromatography (HPLC) method for quantification of atropine sulphate and OB in the antidote solutions was developed and in‐house validated.

## 2 METHODS

### 2.1 Chemicals and standard solutions

AT (certified reference material) and OB (≥95.0%, HPLC grade), were supplied from Sigma Aldrich (Schnelldorf, Germany). LC‐MS grade acetonitrile, water and formic acid (FA) were purchased from Merck (Darmstadt, Germany).

### 2.2 Standard solutions and sample preparation

The stock solutions of AT and OB were prepared at the concentration of 1 and 10 mg/ml in water, respectively. Working solutions and calibration standards were prepared by diluting the stock solutions with water.

The loss of injection volume was assessed by triggering the autoinjector into 100 ml volumetric flasks. The respective weight of the solution was converted into the volume using a density. The density of the antidote solution was determined previously by performing 10 measurements for AT, OB and mixed solutions using a pycnometer. The samples were then diluted with water, 1:25 (v:v), divided into triplicates and analyzed on HPLC for the quantitative assessment of the two drugs in the antidote solutions.

### 2.3 Autoinjector

DOUBLEPEN OA (ChemProtect, Czech Republic) is a two‐chamber autoinjector, containing OB with a declared concentration of 220 mg/2 ml in one chamber and AT with a declared concentration of 2 mg/2 ml in the other. The aqueous OB and the AT solutions have pH values of 4.8 and 2.5, respectively. Both solutions contain the excipients sodium chloride, disodium EDTA, benzyl alcohol, and glycine to increase stability. The autoinjector has a length of 140 mm and its maximum diameter is 24 mm. The two‐chamber reservoir filled with antidote solutions is 70 mm long and has a maximum diameter with a closure of 20 mm. The weight of the autoinjector is 70 g and the needle has a length of 20 mm. The antidote solution should be administered intramuscularly as soon as intoxication is detected. To trigger the autoinjector one has to remove the metal safety pin, place the autoinjector against a thigh muscle, push the safety fuse for antidote application and hold for 10 seconds. After the application one should remove the autoinjector from the muscle. The storage conditions are dark and dry place at a temperature of 5–25°C.

### 2.4 HPLC analysis

The samples for quantitative analysis, prepared according to Section 2.2, were analyzed on the Nexera XR UHPLC system (Shimadzu, Austria, DGU‐20A5R Degassing unit, LC‐ 20ADXR Liquid Chromatograph Pump, SIL‐20AXR Autosampler with 0.1 mm stainless steel tubing, SPD‐M20A Prominence Diode Array Detector with a cell volume of 10 µl, a data collecting frequency of 40 Hz and a response time of 25 ms, CTO‐20AC Prominence Column Oven).

Reversed‐phase chromatographic separation was performed on Primesep C HPLC column (3 µm, 3.2 × 50 mm, Sielc Technologies, USA), equipped with a suitable precolumn, and at a flow rate of 0.4 ml/min. The mobile phase for isocratic HPLC analysis was water containing 0.1% FA/acetonitrile containing 0.1% FA (92:8, v:v). The injection volume was 1 µl for all samples. The analytes were observed with Photodiode Array Detector (PDA) at the two different UV wavelengths, at 210 and 280 nm for AT and OB, respectively. The column compartment was maintained at 40°C.

### 2.5 Method validation

The optimized method was validated using International Council for Harmonisation (ICH) Q2(R1) guidelines.^19^ The following validation parameters were determined: linearity, accuracy, precision, the limit of quantification (LOQ) and the limit of detection (LOD). The linearity was evaluated across the concentration range of 500–4000 mg/L for OB and 10–90 mg/L for AT by analyzing triplicates of calibration standards on three successive batches. The calibration curves were generated by plotting the peak area from an analyte against the initial concentration. Accuracy, repeatability, and intermediate precision were estimated from the data points obtained from calibration curves. The difference between calculated and true concentration was defined as accuracy, while precision was expressed as coefficient of variation (%CV). Repeatability was calculated by measuring prepared calibration standards three times within the same batch, while intermediate precision was calculated by analyzing the same set of samples on twelve separate days. The accuracy and precision were limited to be within ±15% of the nominal concentration, except at the LOQ, where it should be within ±20%. Both LOD and LOQ were calculated by dividing the standard deviation of the lowest calibration standard by the square root of replicated measurements (*n* = 9) on this level and multiplied by 3 and 10, respectively. The matrix effect of AT was quantitatively assessed by dividing the average peak area of the analyte in standard solution in water by the average peak area of standard solution in water containing all the excipients mentioned in Section 2.3 with the analyte at the same concentration and multiplied by 100. The matrix effect was found to be insignificant.

### 2.6 Uniformity of dosage units

The 98 samples of the two production batches, 1707068 and 1707067, were tested on uniformity of dosage unit by content uniformity method according to chapter 2.9.40 of the European Pharmacopoeia.^20^ For the method to be acceptable not less than 30 units must be selected. The preparation of each unit was conducted according to the instructions for content uniformity of liquid dosage forms. Each batch of 10 units was individually assayed. Acceptance value (AV) was calculated using Equation (1).^20^

(1) $$AV=|M-\bar{X}|+ks$$

where $\bar{X}$ is a mean of individual contents (*x*_1_ *, x*_2_ *,…, x*_n_), calculated from 10 individual contents of the dosage units tested and expressed as a percentage (%) of the label claim, *M* is the reference value, *k* is the acceptance constant which is 2.4 for 10 units and *s* is the standard deviation of the sample. The percentage of the label claim was calculated from individual content divided by the label claim from the manufacture and multiplied by 100. The requirements for dosage uniformity are met if the AV of the first 10 dosage units is less than or equal to 15.0%.^20^

## 3 RESULTS AND DISCUSSION

### 3.1 HPLC analysis

**FIGURE 2:**
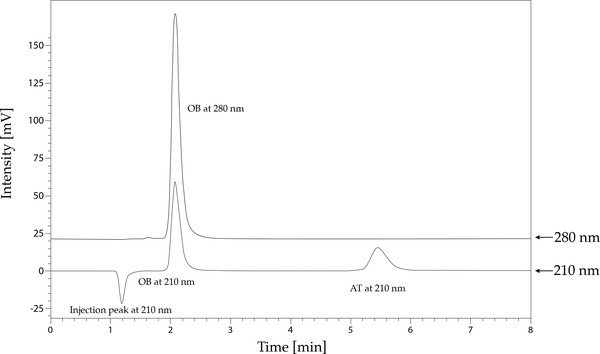
A chromatogram of 100 mg/L of AT and OB standard solution. Chromatographic elution was monitored at 280 nm with a retention time of 2.1 min for OB and at 210 nm at 5.5 min for AT. AT ‐ atropine sulphate monohydrate, OB ‐ obidoxime dichloride

A simple HPLC method was developed for the quantification of AT and OB in the injection solution. Figure 2 shows the chromatographic separation of the two analytes at the optimal HPLC conditions. Since the substances under investigation gave a different absorption maximum the analysis was conducted at two different wavelengths, at 210 nm for AT and 280 nm for OB. Due to the high polarity of OB and its interaction with the silica matrix of regular reversed‐phase columns, the Primesep C column was used. The carboxylic groups on the silica surface of the column shield residual silanols from interacting with a quaternary amine. OB is retained on the column by strong cation exchange and weak reverse phase mechanisms, thus allowing a simultaneous HPLC determination of AT and OB without complex and time‐consuming sample preparation.

### 3.2 Method validation

The HPLC method was validated according to ICH Guidelines by assessing linearity, accuracy, precision, LOQ and LOD.^19^ As can be seen in Table 1, overall, the validation was successful. Calibration curves showed linearity for the concentration ranges over the extent of 500–4000 mg/L for OB and 10–90 mg/L for AT with regression coefficients of 0.9996 and 0.9994, respectively. Each of the concentration standards was prepared in triplicates and injected three times to get a repeatable response. The linear regression equation for calibration curves was y = 13.503x – 239.21 for OB and y = 2.5600x – 6.9153 for AT, where y indicates the peak area and x represents the concentration in mg/L. The results for accuracy contain the relative error (RE%) which was obtained from calculated and true values of the calibration standard (Table 1). The calculated RE% ranged from 98.3‐102.3% and 98.2‐107.0% for OB and AT, respectively. The repeatability was 0.4‐3.2% for OB and 0.8‐3.0% for AT, and the intermediate precision was 2.7% for OB and 0.7% for AT. The obtained values for accuracy and precision are all within ±15% of the nominal concentration, indicating that the method is accurate and reproducible.^19^ Reasonable LOD and LOQ levels, calculated as described in Section 2.5, were achieved. The LOD of the presented method was 3 mg/L for OB and 0.3 mg/L for AT, whereas the LOQ was 10 mg/L for OB and 1 mg/L for AT.

**TABLE 1:** Method validation parameters for obidoxime dichloride (OB) and atropine sulphate monohydrate (AT) including concentration range, regression coefficient (R^2^), accuracy, repeatability, intermediate precision, the limit of detection (LOD), the limit of quantification (LOQ) and matrix effect (ME) (*n* = 9)

**Analyte**	**Conc. range*^a^***	**R** ^2^	**Accuracy**	**Repeatability**	**Inter. precision**	**LOD**	**LOQ**	**ME**
	(mg/L)		RE% (%)	%CV (%)	%CV (%)	(mg/L)	(mg/L)	%
OB	500–4000	0.9996	98.3–102.3	0.4–3.2	2.7	3	10	n.e.
AT	10–90	0.9994	98.2–107.0	0.8–3.0	0.7	0.3	1	103.7

AT ‐ atropine sulphate monohydrate.

OB ‐ obidoxime dichloride.

%CV ‐ coefficient of variation.

RE% ‐ relative error.

*^a^* Calibration curve contained 8 and 9 calibration standards for OB and AT, respectively.

n.e. – not evaluated.

### 3.3 Evaluation of the autoinjector samples

To obtain significant results a total of 100 autoinjectors were analyzed. Prior to the investigation, two autoinjectors were disassembled to determine the exact content in each chamber. Interestingly, the quantitative analysis of the disassembled autoinjectors indicated higher content of both drugs in the two‐chamber reservoir. The actual concentration of OB was found to be 60 and 56 mg/ml which is 110% and 102% of the label claim. For AT, the content of 0.6 and 0.7 mg/ml was found to correspond to 124% and 135% of label claims (Table S1).

The remaining 98 samples of the two production batches 1707068 and 1707067 were analyzed to investigate possible inconsistencies and/or variations in volume and content after triggering the autoinjector. The results of quantification for AT and OB in the antidote solution are calculated for each autoinjector according to its administration volume. The density of the autoinjector antidote solution was 1.0225 g/ml at room temperature (Data not shown). After triggering the autoinjector, the average loss of the actual injected volume was an average of 5% (Table S2). This result has further strengthened our hypothesis that by applying two‐chamber autoinjectors it is highly possible that not all the content is administered. As stated by the producer the content of OB and AT after administration corresponds to 220 and 2 mg, respectively. Indeed, the average administered content for both drugs was found to be slightly lower than stated by the producer in batch 1707068. The median content after injection of OB and AT for batch 1707068, were 216.5 and 1.9 mg, respectively (Table 2). Analysis of batch 1707067 revealed a slightly higher median content of OB (223.9 mg) than stated by the producer, while the median content of AT was slightly decreased as in batch 1707068, namely 1.9 mg (Table 2).

**TABLE 2:** Content of obidoxime dichloride (OB) and atropine sulphate monohydrate (AT) in two‐chamber DOUBLEPEN OA autoinjector (total of 2 ml per chamber) from two production batches 1707068 (*n* = 50) and 1707067 (*n* = 48)

	**OB**	**AT**
Batch 1707068 (*n* = 50)		
Average (mg)	216.5	1.9
Median (mg)	216.5	1.9
Minimum (mg)	200.8	1.7
Maximum (mg)	226.9	2.1
Average (% of label claim)	98	96
Batch 1707067 (*n* = 48)		
Average (mg)	224.2	2.0
Median (mg)	223.9	1.9
Minimum (mg)	208.5	1.8
Maximum (mg)	234.6	2.2
Average (% of label claim, %)	102	99

AT ‐ atropine sulphate monohydrate.

OB ‐ obidoxime dichloride.

**FIGURE 3:**
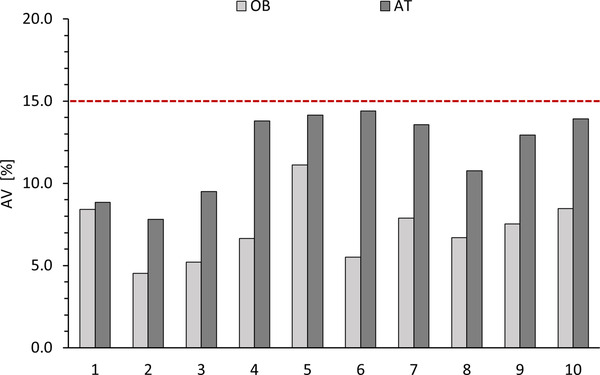
Calculated AV values of OB and AT for the batch 1707068 (1–5) and 1707067 (5–10) in the antidote solutions of the DOUBLEPEN OA two‐chamber autoinjector. Each batch of 10 units was individually assayed (1–10). The solid line indicates the requirements of the European Pharmacopoeia (AV ≤ 15%). AT ‐ atropine sulphate monohydrate, OB ‐ obidoxime dichloride, AV ‐ acceptance value

According to chapter 2.9.40 of European Pharmacopeia^20^ for the assessment of content uniformity for liquid dosage forms 10 units were assayed individually using the in‐house validated HPLC method. The units were categorized into groups, thus each group containing 10 units. The calculated % of label claim for individual units were used for the calculation of the average $\bar{X}$ for each group. The AV values, calculated according to Equation (1), are shown in Figure 3. To assess the AV following criteria were applied: The requirements for dosage uniformity are met if the AV of the first 10 dosage units is less or equal to the maximum allowed acceptance value (*L1*), which is according to European Pharmacopeia 15.0.^20^ The AV value of the first 10 dosage units for the batch 1707068 (Figure 3, group 1) was less than 15.0%. Even if the AV value of the first 10 dosage units for batch 1707067 (Figure 3, group 5) showed the highest values in the conducted test, it was still found to be below 15.0%. According to these findings, both production batches have met the requirements of uniformity of dosage unit by the European Pharmacopeia.^20^

## 4 CONCLUSIONS

In this work, we report the successful application of an HPLC method for the quantification of AT and OB in the antidote solutions of the DOUBLEPEN OA two‐chamber autoinjector system. The method was proved to be simple, accurate and repeatable. According to our results, both production batches have shown a loss of volume and content of antidote solutions after triggering the autoinjector. Nonetheless, it is important to emphasize that the loss of the content was within European Pharmacopoeia requirements in terms of the uniformity of dosage units.^20^

## AUTHOR CONTRIBUTIONS

Conceptualization, Judith Wackerlig and Richard Wosolsobe; experiments and analysis, Paniz Morawej and Rainer Stinzl; validation, Paniz Morawej; investigation, Judith Wackerlig; data curation, Iva Spreitzer; writing—original draft preparation, Iva Spreitzer; writing—review and editing, Iva Spreitzer, Judith Wackerlig and Richard Wosolsobe; project administration, Judith Wackerlig; funding acquisition, Richard Wosolsobe. All authors have read and agreed to the published version of the manuscript.

## CONFLICT OF INTEREST

The authors declare that they have no conflict of interest.

## Supporting information



Supporting Information

## Data Availability

The datasets generated and analysed during the current study are available from the corresponding authors upon reasonable request.
