# Supplementary material for: Quantification of atropine sulphate monohydrate and obidoxime dichloride in two‐chamber autoinjectors for accessing uniformity of dosage
Source: Anal Sci Adv. 2022 Nov 12;3(11-12):297–303. doi: 10.1002/ansa.202200028 (PMC10989631; doi:10.1002/ansa.202200028)
Supplement: Supplementary file 1 — Supporting Information [file ANSA-3-297-s001.docx]

Supplementary Materials: Quantification of atropine sulphate monohydrate and obidoxime dichloride in two-chamber autoinjectors for accessing uniformity of dosage

Iva Spreizter^1^, Paniz Morawej^1^, Richard Wosolsobe^2^, Rainer Stinzl^3^ Judith Wackerlig^1,*^

^1^Department of Pharmaceutical Sciences, Faculty of Life Sciences, University of Vienna, Vienna, Austria.

^2^Military Pharmacy Section, Medical Division, Federal Ministry of Defence, Vienna, Austria

^3^Armaments and Defence Technology Agency, Vienna, Austria

*Correspondence: judith.wackerlig@univie.ac.at

**Table S1** Concentration of OB and AT in the autoinjector chambers before injection as pure substances (actual) compared to the concentrations specified by the manufacturer (target) and their respective percentage of label claim.

|  | OB | AT |
| --- | --- | --- |
| **Sample No. 1** |  |  |
| Actual conc. (mg/mL) | 60 | 0.6 |
| Target conc. (mg/mL) | 55 | 0.5 |
| % of label claim (%) | 110 | 124 |
| **Sample No. 2** |  |  |
| Actual conc. (mg/mL) | 56 | 0.7 |
| Target conc. (mg/mL) | 55 | 0.5 |
| % of label claim (%) | 102 | 135 |

AT - atropine sulphate monohydrate; OB - obidoxime dichloride.

**Table S2** Table S2 Volume loss after the application of DOUBLEPEN OA autoinjector from two production batches 1707068 (n=50) and 1707067 (n=48). The volume loss was calculated by weigh chambers with antidote solution, cleaned chambers and antidote so-lution after application in volumetric flask.

|  | Volume loss (%) |
| --- | --- |
| **Batch 1707068 (n=50)** |  |
| Average (%) | 5.1 |
| Median (%) | 5.1 |
| Minimum (%) | 2.9 |
| Maximum (%) | 8.9 |
| **Batch 1707067 (n=48)** |  |
| Average (%) | 5.3 |
| Median (%) | 5.1 |
| Minimum (%) | 2.5 |
| Maximum (%) | 16.4 |
